# Mechanical shear controls bacterial penetration in mucus

**DOI:** 10.1038/s41598-019-46085-z

**Published:** 2019-07-04

**Authors:** Nuris Figueroa-Morales, Leonardo Dominguez-Rubio, Troy L. Ott, Igor S. Aranson

**Affiliations:** 1 Department of Biomedical Engineering, The Pennsylvania State University, University Park, PA 16802 USA; 2 Department of Animal Science and Center for Reproductive Biology and Health, and the Huck Institute for the Life Sciences, The Pennsylvania State University, University Park, PA 16802 USA

**Keywords:** Fluids, Bacteria

## Abstract

Mucus plays crucial roles in higher organisms, from aiding fertilization to protecting the female reproductive tract. Here, we investigate how anisotropic organization of mucus affects bacterial motility. We demonstrate by cryo electron micrographs and elongated tracer particles imaging, that mucus anisotropy and heterogeneity depend on how mechanical stress is applied. In shallow mucus films, we observe bacteria reversing their swimming direction without U-turns. During the forward motion, bacteria burrowed tunnels that last for several seconds and enable them to swim back faster, following the same track. We elucidate the physical mechanism of direction reversal by fluorescent visualization of the flagella: when the bacterial body is suddenly stopped by the mucus structure, the compression on the flagellar bundle causes buckling, disassembly and reorganization on the other side of the bacterium. Our results shed light into motility of bacteria in complex visco-elastic fluids and can provide clues in the propagation of bacteria-born diseases in mucus.

## Introduction

Mucus is a viscoelastic gel coating the surfaces of cells and tissues in animal organ tracts exposed to the external environment^1^. Although permeable to oxygen and nutrients, it serves as a front-line protection against invasion and colonization of tissues by pathogens^2–4^. Sexually transmitted infections occur when bacteria and viruses traverse the mucus of the female reproductive tract. Respiratory diseases like cystic fibrosis and asthma are associated with abnormal mucus rheology. In general, mucus plays an exceptionally wide range of important biological roles^5^, mostly related to allowing selective entry or exit of substances into the body while inhibiting invasion by pathogens. It is believed that rheology and anisotropy of flowing mucus is important for creating privileged pathways for sperm transport and “filtering out” abnormally shaped sperm^6^.

The macromolecular components of mucus include plasma-derived and locally-produced proteins and a polydispersed population of high molecular weight glycoproteins, the mucins. These components form a supramolecular network with emergent properties, such as complex viscoelastic responses^7^, resulting from their diverse interactions^8^. Depending on the concentration and composition of mucin, mucus can behave as a liquid crystal^9–15^.

On a microscopic scale, mucus can be viewed as a highly heterogeneous cross-linked dense polymer network^16,17^. The polymer fraction constitutes only about 5–10% of the components of mucus^18^. The pore size for bovine cervical mucus was estimated as ~13 *μm* in^19^. These estimates are is in agreement with confocal microscopic^20^ and electron microscopic^21^ measurements, determining pore size in the range 1–20 *μm*. These dimensions are comparable with the typical dimensions of bacteria (body length ~4 *μm*, flagellar length ~10 *μm*, flagellar thickness ~20 *nm*). Thus, bacteria will be swimming in a heterogeneous environment determined by the configuration of the mucus polymer network. Furthermore, the mesoscale (neither micro or macro) rheology of mucus will strongly affect the bacterial motility.

While the individual behavior of bacteria in Newtonian fluids (e.g., water) is fairly well established, a fundamental issue is to understand how mucus anisotropic organization and visco-elasticity affects the behavior of swimming bacteria and their interaction with shear flow and surfaces. This problem is relevant, for example in the context of bacteria-caused intestinal infections. *Helicobacter pylori* bacteria are a main cause of chronic atrophic gastritis, an inflammatory precursor of gastric adenocarcinoma, the world’s second most common cancer^22^. To penetrate the protective mucus layer, *Helicobacter pylori* generate ammonia that locally transforms nearby mucus gel into a fluid pocket^23,24^. Invasion and degradation of mucus layer in the reproductive tract by pathogenic bacteria may also cause female infertility^25^.

By performing *in vitro* studies of *Escherichia coli* and *Bacillus subtilis* motility in cervical mucus, we established that mucus anisotropy and heterogeneity depends on how the external mechanical stress is applied. In experiments in shallow mucus films we observed “bouncing” bacteria reversing their swimming direction by 180 without making U-turns. Furthermore, we observed that the reverse motion is systematically faster by 20–30% than forward motion. We associate this increase of the reverse speed with the formation of a transient tunnel made by the bacterium during forward movement. These tunnels lasted for several seconds enabling the bacteria to swim back faster than in initially unperturbed mucus. Taking advantage of high-resolution fluorescent microscopy and automated tracking of multiple bacteria, we elucidated the mechanism for the direction reversal by fluorescent labeling of the bacterial flagella. We demonstrated that when the bacterial body is suddenly stopped by the mucus structure, the rotation of flagella induces a significant compression on the flagellar bundle and causes it to buckle, disassemble and reorganize on the other side of the bacterium. Our results shed light on how stress-dependent organization of complex visco-elastic fluids exemplified by mucus affects bacterial motility. In addition, our study can possibly provide clues to bacterial invasion of mucosal surfaces of the gastric, respiratory and genital tracts.

## Experiments and Results

### Invasion of mucus by bacteria

#### Parallel plates

**Figure 1:**
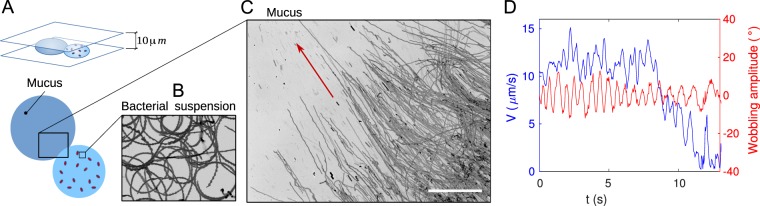
Bacteria invasion of mucus. (**A**) Schematics of the experiment: a droplet of mucus in contact with a droplet of bacteria-rich TB is sandwiched between two parallel glass slides. (**B**) Trajectories of Bacillus subtilis (strain 1085) obtained in the region with TB, by consecutive snapshot accumulation over 8.5 seconds. The bacteria display the well known circles close to a wall. The image is (125 × 94) *μm*. (**C**) Trajectories of bacteria over 8.5*s* at the interface of the droplets. The bacteria penetrate in the direction of the red arrow. Scale bar 100 *μm*. (**D**) Velocity and wobbling amplitude of a bacterium penetrating mucus.

We performed *in vitro* studies of bacterial invasion of cervical mucus. First, we examined the interface between a droplet of mucus and a droplet of bacterial suspension (e.g. *Bacillus subtilis* in a Newtonian fluid such as Terrific Broth (TB) growth medium, see Fig. 1(A) and Supplementary Video V1). The liquid droplets were confined between two glass slides separated by a 10 *μm*-thick double-sided tape acting as a spacer. The spacer also sealed the sample and prevented evaporation.

In a homogeneous liquid environment exemplified by Newtonian fluids such as water or TB, bacteria display erratic trajectories resulting from their run-and-tumble process and Brownian rotational diffusion^26,27^. Differently, bacteria swimming close to solid boundaries in quiescent fluids move in circular trajectories^28^ as a result of their body and flagellar counter-rotation. Here, we recover the expected circles when monitoring the dynamics in the drop of TB, which we display with a superposition of snapshots in Fig. 1(B). Strikingly, at the interface between TB and mucus, the circles do not longer take place and the bacteria advance parallel towards the region with mucus. This organized invasion is evident in panel (C), in a similar superposition of snapshots. The paths are abnormally straight lines that extend for as long as 400 *μm*. Moreover, the parallel paths were only present after squeezing the drop of mucus. When gently depositing a drop of mucus inoculated with bacteria, their trajectories are randomly oriented. A movie in Supplementary Materials (Video 2) further illustrates this points.

The parallel bacterial invasion suggests the existence of anisotropic properties in the mucus when it is sheared, making the radial direction preferential for bacterial locomotion. The experiment was repeated using different types of bacteria: *Bacillus subtilis* strains DS1919 (run-and-tumble) and 1085 (non-tumbler mutant), as well as *Escherichia coli* strains RP437 (run-and-tumble) and CR20 (non-tumbler mutant). In all cases we obtained qualitatively similar phenomena. Transversal deviations of the swimming direction were rare, but more often observed in the regions that were previously traversed by many other bacteria, indicating that bacteria alter the local anisotropic organization of the mucus polymer network.

The average swimming velocity drastically drops while swimming in mucus. As bacteria penetrate into the gel, the surrounding fluid becomes more and more viscous and the motion is slowed, as shown in Fig. 1(D). Both velocity and orientation of the bacterial body exhibited oscillations with a period of 0.6*s* (wobbling). These oscillations are due to the precession of the bacterial body, e.g. due to misalignment of the flagella bundle with the bacterial body, similar to that observed in Newtonian fluids^29^. As bacteria advanced deeper into mucus, the wobbling period increased, likely due to a gradual viscosity increase of the water-mucus mixture.

**Figure 2:**
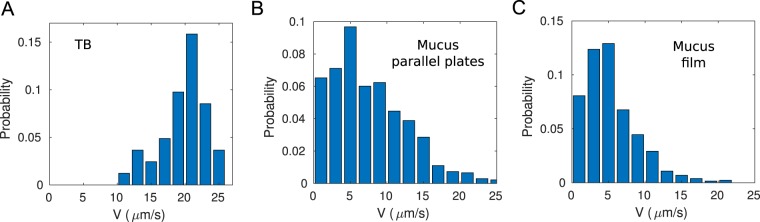
Distributions of average velocities of *Bacillus subtilis* bacteria (strain 1085). (**A**) Histogram for bacteria swimming in TB. (**B**) Histogram at the interface of two mixing droplets, see Fig. 1. (**C**)Histogram for bacteria swimming in a thin film of mucus.

We measured the macroscopic rheology of mucus (see Supplementary Information) and found it in agreement with previous studies^30–32^. The complex viscosity of mucus was found to be of the order of 0.1 *Pa s* for the typical shear rates of bacterial swimming (1*s*^−1^), which is two orders of magnitude higher than the viscosity of water. However, bacteria are still capable of swimming in this medium at velocities only slightly smaller than in water. Figure 2 shows the distributions of average velocities of bacteria swimming in TB, between parallel plates at the interface TB-mucus and in a thin film of mucus. Even when the bacterial velocities in mucus could be as low as 1 *μm*/*s*, their invasion persisted with ballistic trajectories. The mechanisms underlying the bacterial ability to swim in conditions of high visco-elasticity, like the ones in these experiments, are an active field of research^33,34^. Furthermore, bacteria were sometimes trapped in place as if their flagella were tethered by the mucus.

#### Thin films

**Figure 3:**
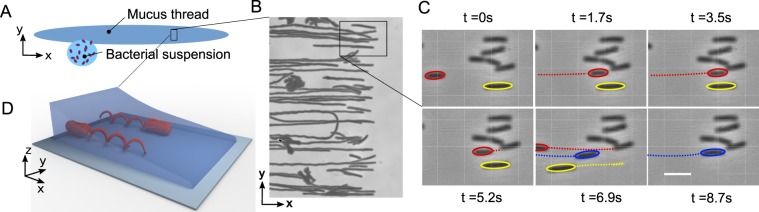
Reversal direction in a thin film. (**A**) Schematics of the experiment: a thread of mucus deposited in a glass slide is put in contact with a droplet of bacteria-rich TB. We visualize the dynamics of bacteria in a shallow part of the mucus film. (**B**) Superimposed snapshots showing bacterial trajectories as they arrived to the front, bounced and swam back. Only a few bacteria made a U-turn, most of them reversed motion direction. (**C**) Sequence of snapshots over 2*s* (blowout of panel (B)) showing the bacterium (marked red) arriving at the front, reversing direction and swimming back without U-turn. Scale bar 5 *μm*. (**D**) Sketch of bacteria swimming towards and leaving a shallow meniscus of mucus.

Secondly, we investigated bacterial invasion of a thin film of mucus. This situation models, for example, bacterial motility in thin mucus layers protecting epithelial cells. In this experiment, a droplet of the bacterial suspension (Bacillus subtilis) was placed next to a shallow thread of mucus in a chamber that prevented evaporation. Figure 3(A) depicts the experimental configuration. The thread was deposited using a 30 gauge needle attached to a syringe. The cover-slip was not in contact with the liquids and the experiment occurred under thin-film conditions, i.e., a glass substrate and a liquid-air interface. Bacteria moved from the droplet of TB to the film of mucus and continued swimming parallel to the mucus thread.

Figure 3(B) displays projections of bacterial positions in the film over 10 s. This observation highlights a novel phenomenon: a 180 reversal of the bacterial swimming direction induced by the geometrical constraints in the mucus film. Supplementary Video 3 illustrates the phenomenon. Note that the direction reversal could also take place as a U-turn, resembling a car turning back in the direction from which it has come. Figure 3(B) shows a single U-turn among many 180 direction reversals. Interestingly, U-turns are rather rare (about 4–5% of total reversal events) and most of the swimmers return without any rotation of their bodies.

**Figure 4:**
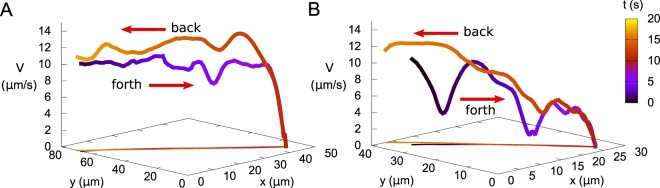
Motion reversal on the same track. Panels (A) and (B) show trajectories and velocities of two different bacteria bouncing from the front. Bacteria leave the bouncing point faster than they approached it.

Select bacteria positions are shown in Fig. 3 (C) in a sequence of consecutive snapshots for every 1.7*s*. It clearly demonstrates the swimming direction reversal for the cell labeled in red. This effect is not associated with the run-and-tumble behavior, since we consistently observed it for smooth-swimming bacteria (e.g. the bacterial strain that tumbles very rarely), and therefore, it is different from the direction reversal taking place in liquid crystals^35^. The reversal phenomenon bears some similarity with the reversal of bacterial swimming direction at an obstacle observed in Newtonian fluids^36^. However, the seeming resemblance is rather incomplete. In contrast to Newtonian fluid in ref.^36^, in mucus the reversing bacteria closely trace their own tracks. Furthermore, the backward motion is significantly faster than the forward one (see Fig. 4).

We observed some rare events of bacterial reversals in the bulk of unsheared mucus. We associate this events to the presence of inhomogeneous regions in the mucus, producing the scattering of the cells. In those cases bacteria did not closely trace back their tracks, much as the phenomenon reported in^36^.

Note that in Fig. 3(B) all bacteria arrive at a well-defined front. This front moved forward at a mean speed of 0.17 *μ*m/s, but in general, the speed changes depending on the thickness of the mucus layer. Under these conditions, the bacteria stopped momentarily at the front prior to swimming backward. The waiting times were on average 12 seconds, but some remained motionless at the border for times as long as 40 *s*, until motion was triggered by newly arriving bacteria. In general, the reversal takes place in the proximity of very shallow parts, such as the borders of films and drops, where the film of mucus becomes sufficiently thin and bacteria cannot continue moving along it, as sketched in Fig. 3(D).

In most of the cases, bacteria leave the front following the same path that they used for approaching it. It is shown in Fig. 4(A,B), presenting the *x*,*y*-coordinates and velocity in the vertical axis of two bacteria approaching and leaving the front. The color code indicates time. The projection shows the superposition of the forward and backward trajectories on the same straight line. Moreover, between 10 to 50 *μm* near the front, the velocity during backward motion was in average 20% higher than the approaching velocity at the same *x*,*y*-position (forward velocity (8.4 ± 2.8) *μm* vs backward velocity (10.4 ± 2.4) *μm*). The results suggest that the bacteria create transient tunnels in mucus and these tunnels last for at least a few seconds. When the backward tracks deviated considerably from the forward tracks, the velocities in both directions were roughly the same.

**Figure 5:**
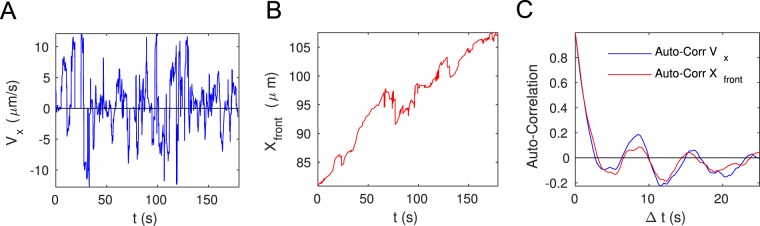
Cooperative dynamics at the interface. (**A**) Average velocity of bacteria in a thin film behind the front. (**B**) Advancing position of the front. (**C**) Autocorrelation of average velocity and of the position of the front, showing clear periodicity.

Apparently, the bacteria reversals at the front are not completely random. The reversals have a well-defined period of about 8 sec. As one can see from Fig. 5(A), the average velocity of the swimmers along the main axis of the meniscus displays well-pronounced periodic oscillations. In SI we show the power spectrum of the average velocity, with the main peak at 0.13 Hz, which is consistent with the collective reversal period of about 8 sec. When analyzing the auto-correlation of the numbers of bacteria arriving or leaving the interface in the unit time, this periodicity is not present. Neither individual velocities of bacteria show a stop-and-go dynamics. The phenomenon is rather complex instead, as is has to do with the interplay between the rheological properties of mucus, surface tension and thickness of the film, and probably the bacterial concentration as well. When analyzing the motion of the front, which position we define as the average *X* coordinate of the 10 most advanced bacteria, we find a periodic advance, together with repetitive withdrawals (Fig. 5(B)). It is quite likely that pinning and depinning of the contact line gives rise  to the oscillations in the average velocity, as we can see from the close agreement between their auto-correlation functions shown in panel (C).

### Alignment of passive objects

**Figure 6:**
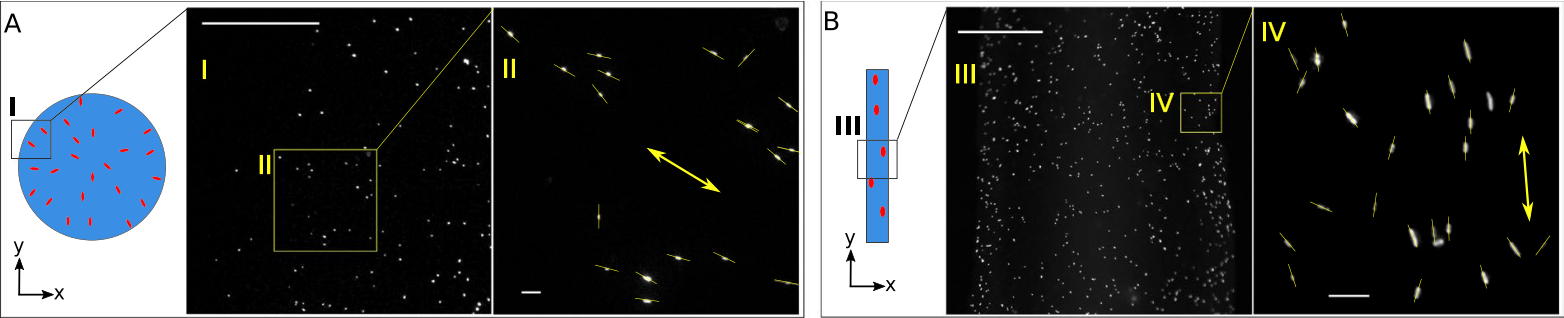
Alignment of passive particles in mucus. (**A**) Droplet is squeezed between two parallel glass slides separated by 10 *μm*. The tracers align radially. Scale bars 200 *μm* (I) and 10 *μm* (II). (**B**) Mucus thread is deposited on a cover slip with particles aligned parallel to the thread. Scale bars 200 *μm* (III) and 10 *μm* (IV) correspondingly.

To understand the origin of the alignment of swimming bacteria in mucus, we suspended non-motile fluorescent bacteria in endo-cervical mucus and deposited it on a glass slide. Flagella were cut by multiple pipetting so the bodies could be used as elongated tracer particles. When the sample was deposited as a droplet and squeezed between parallel plates, as in Fig. 6(A), the particles align radially. The average orientation direction is represented by a yellow arrow. When mucus is deposited as a thread on a cover slip, as in panel (B), the particles align parallel to the main axis of the thread. Typical time for Brownian disorientation of the particles of this size is estimated to be 0.8 seconds, obtained for a rigid ellipsoid of length 2 *μm* and radius 0.4 *μm* in water at temperature *T* = 25 °C. In our experiment, the bodies stayed aligned for the entire duration of the experiment (several minutes). Water constitutes a high fraction of the components of mucus (~95%)^18^, therefore, it is the microstructure of mucus that determines the dynamics of diffusion in this medium. These observations hint that mucus becomes an effective anisotropic medium when subjected to shear stress. The anisotropic structure of mucus is preserved for very long times, thus preventing the reorientation of the bacterial bodies. We obtained similar orientations for gold rods, but their visualization and automatic detection was more challenging, due to a heterogeneity of the mucus gel.

### Cryo SEM characterization of mucus

**Figure 7:**
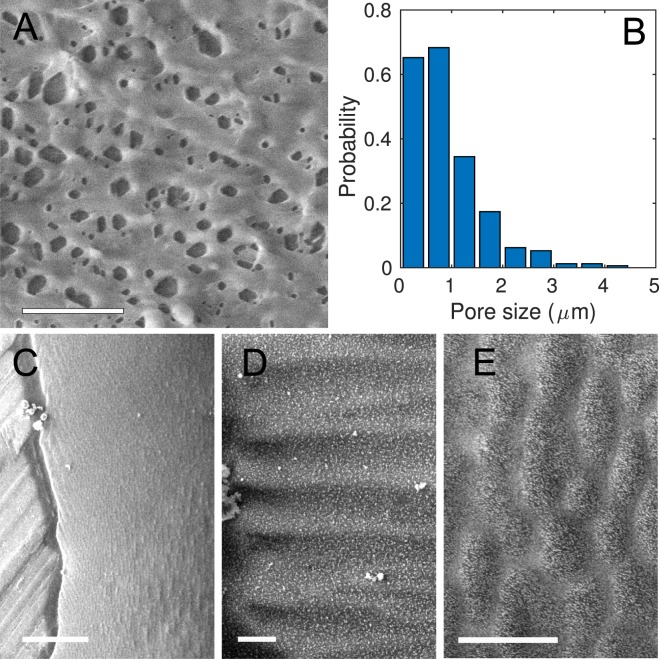
Cryo SEM images of mucus. (**A**) Image of a drop showing a pore mesh. Scale bar 10 *μm*. (**B**) Histogram of pore sizes in mucus, as extracted from panel (A). (**C**) Mucus thread deposited vertically. Scale bar 100 *μm*. There is a vertical alignment of the mucus micro-structure, parallel to the orientation of the thread. The borders, however, show wrinkles almost perpendicular to the main direction. (**D**) Wrinkles at the edge of the thread, oriented perpendicular to the walls. Scale bar 10 *μm*. (**E**) Blowup of (**C**) showing the main vertical alignment close to the center of the thread. Scale bar 10 *μm*. Some ice crystals are present in panels (C) and (D).

To gain insight into the consequences of mechanical stress on the structure of mucus, we performed Cryo Scanning Electron Microscopy (cryo SEM) observations of mucus samples (see Methods for details). The results are summarized in Fig. 7. The micrographs were taken without any deposition of conductive elements on the surface, therefore, preserving the microstructure of the sample. Panel (A) focuses on a drop that was not strained. The drop displays a mesh structure with the pores of a few microns in size. The average pore size in this figure is 0.9 *μm*, with a standard deviation 0.7 *μm*. The distribution of pore sizes is displayed in panel (B). The size measurements are the major and minor axes lengths extracted from detection of the holes as ellipses. The rest of the panels focus on a thread deposited vertically on the holder. Panel (C) shows the substrate on the left and the frozen mucus on the right. The borders of the thread are irregular and display a wrinkle-like structure that forms a certain angle with the border depending on the region. A blowup of these wrinkles, aligned perpendicular to the lateral border, is shown in panel (D). Far from the border, the mucus shows features aligned mostly parallel to the borders. These can be seen in more detail in panel (E). The typical sizes of these features change depending on the region, and possibly also with the local stress. The smallest pores present in the droplet without tension are not visible in the stretched mucus. These observations demonstrate that mechanical stresses alter the local spatial arrangement of the protein complexes forming the gel structure.

## Discussion

Motility and self-organization of active self-propelled particles in anisotropic and visco-elastic media is an active area of research^35,37–43^. Moreover, understanding the interaction between bacteria and mucus is key to developing better ways to fight pathogen invasion at mucosal surfaces.

The ability of microorganisms to move through gel-like materials is determined by the shape and mechanical properties of their body and flagella, as well as the fluid rheology^44^. Due to a substantially increased average viscosity of the concentrated mucus medium, the shear flow will exert much higher forces on the flagella potentially resulting in significant bending. Bending of flagella, in turn, will lead to the reorientation of a bacterium and potentially very different interactions at the boundaries as compared to Newtonian fluids^45,46^.

**Figure 8:**
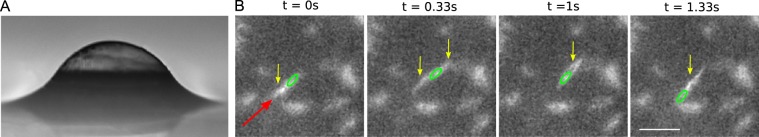
Bouncing of bacteria in a shallow meniscus. (**A**) Side view of a mucus droplet showing a wide shallow precursor film where the bacteria bounce. The height of the droplet is 100 *μm*. (**B**) Sequence of fluorescent images illustrating flagella reorganization. The bacterial body is highlighted with a green oval. Initially, the bacterium was swimming in the direction of the red arrow, then, the body stops and the flagella reorganize on the other side of the body, reversing the swimming direction. The yellow arrows point out the position of the flagellar bundles. Scale bar 10 *μm*.

An interesting phenomenon here found is the bacterial motion reversal that occur following geometrical confinement. The droplets of mucus have wide shoulders, ending in very shallow regions, that appears as a meniscus (see Fig. 8(A)). Gradients of height also take place when depositing a long stripe of mucus. These films can be thinner than the width of the bacterial body. The swimming direction of bacteria usually coincides with the direction of the height gradient. Once the bacteria arrive at a sufficiently shallow region, their bodies get stuck so forward movement ceased. At this point, the swimming direction reverses by 180, almost without U-turns, much like the bouncing of a ball. Note that at these low Reynolds number (10^−5^), inertial effects are negligible and such a motion reversal is only possible if the bacteria are self-propelled while leaving the point of reversal. In other words, the flagella need to disassemble from the bundle and form a new bundle on the opposite side of the body.

To examine the mechanism of direction reversal in mucus, we stained the flagella for direct visualization and we recorded sequences of high-resolution fluorescence images, such as shown in Supplementary Video 4 (see Methods for dyeing details). Figure 8 shows a sequence of snapshots revealing flagella while a bacterium reverses its swimming direction. The bodies can be suddenly stopped by a geometrical constriction, such as a shallow meniscus or an obstacle in mucus. When the body stops, the flagella quickly reorganize at the opposite side of the body, as expected, resulting in a reversal of the swimming direction.

The reversal times changed considerably depending on the geometrical conditions. Bacteria were more likely to get trapped in very shallow films due to heterogeneity of the mucus. In steep height gradients, where the bacteria do not get stuck but reverse almost instantaneously, the flagella reorganization took place on average about (0.6 ± 0.3) seconds. Here the uncertainty is given by the standard deviation of the measured times.

The biological significance of the flagellar bundle disassembly is not clear. Instead of reversing direction, bacteria could keep stubbornly pushing against the meniscus, but this rarely occurs. Typical length of flagella range between 15 and 20 *μm*^47^, which is multiple times the characteristic pore size here determined. The flagella must then undergo conformation changes to re-accommodate on the other side of the body by moving through the mesh of mucus. Flagella can undergo various polymorphic transformations in response to environmental conditions, torsional load, and motor direction reversals^48,49^. This mechanism is exploited by some organisms like the monopolarly flagellated species Shewanella putrefaciens^50^ to reverse direction. From our measurements we note that the flagellar bundles of *Bacillus subtilus* are not tight, (see Supplementary Information), however, steric and hydrodynamic interactions between the filaments^51–53^ does not allow them to bundle individually. Thus, we model the bacterium as a rigid body with a single elastic filament representing an effective flagellum. We propose here that the mechanism for the flagella de-bundling is a result of buckling instability, in some sense similar to that observed for monotrichous marine bacterium *Vibrio alginolyticus*^54^.

For a swimming bacterium, the thrust will be counteracted by the viscous drag forces on the body and along the flagellum, which will be under an internal elastic tension. While swimming, the total thrust developed by the flagellum compensates for the viscous drag acting on the body and on the flagellum^45,46^. When a bacterium hits an obstacle and suddenly stops, the viscous drag acting on the flagellum due to translation of the bacterium disappears. Thus, the thrust will only be compensated by the tension accumulated on the flagellum. Under these conditions, the flagellum could buckle, analogous to self-buckling of a tall column under its own weight^55^. Let us define *F*_*p*_ as the thrust per unit length developed by flagella, *L* is the length of the flagella and *K*_*b*_ is its flexural rigidity. The critical thrust per unit length (${F}_{p}^{c}$) needed for self-buckling obeys the equation

1 $${F}_{p}^{c}=\frac{c{K}_{b}}{{L}^{3}},$$

where *c* = 7.8373^55,56^. Using the numerical values *K*_*b*_ = 3 × 10^−23^*Nm*^2^ ^45^ and *L* = 1.5 × 10^−5^*m* result in ${F}_{p}^{c}=7\times {10}^{-8}$*N*/*m*. This estimation is, however, very sensitive to the flagellar length *L* and the tightness of the bundle.

The thrust developed by the flagella can be estimated from the bacterial swimming speed. The viscous drag acting on the body in water is ~0.3 *pN* and the one on the flagella is also about ~0.3 *pN*, leading to a total drag force ~0.6 *pN*. If we assume that the thrust compensating the total viscous drag is homogeneously distributed along the flagellum, ${F}_{p}^{c} \sim 4\times {10}^{-8}N/m$. This value is comparable with the numerical value obtained for ${F}_{p}^{c}$ from Eq. 1. Moreover, our measurements of bacterial swimming speed hints that the apparent viscosity of mucus solvent is higher than that of water by a factor of two or three. Correspondingly, it leads to a higher value for the thrust force. This suggests that a buckling instability is a plausible scenario under these conditions, possibly constituting yet another example of mechanical failure useful in nature^57^.

The cooperative reversal phenomenon bears a similarity with a stick-slip dynamics. We speculate that the collective escape of bacteria from the front can be related to the intrinsic viscoelasticity of the mucus gel and pinning/depinning of the contact line. Arriving bacteria got stuck at the front and tighten the polymer network of the mucus gel. At some point, the deformations lead to the contact line depinning, local breakdown of the polymer network, its rapid weakening, expansion of the pores and simultaneous release of multiple bacteria.

## Conclusions

Here we used naturally produced endo-cervical mucus to evaluate bacterial movement at mucosal surfaces. We have shown that external mechanical stresses affect the microscopic architecture of mucus gel and force the bacteria to align and swim oriented over very long distances. This indicates an effective nematic (liquid crystalline) organization of the macromolecular components of mucus over macroscopic distances.

Since mucus covers the cervical walls and flows out, its structure must be aligned parallel to the walls. Such an alignment would direct bacteria or sperm cells to swim parallel to the walls^40^, reducing the number of individuals arriving at the epithelial cells. A very high concentration of bacteria would be needed to break the liquid crystalline order of mucus and create a path towards the epithelial cells.

Besides biomedical relevance, our results are likely applicable to a general class of complex fluids exhibiting visco-elastic behavior and long memory effects, both biological or synthetic. Reversals may occur in DNA solution^58^, suspensions of viruses^59^ or long polymers^33^, etc. Our observation that bacteria bounce off the obstacles and follow their own tracks can be used for confining and guiding the bacteria or synthetic microswimmers in microfluidic devices operating with non-Newtonian fluids. Another implication of our study is that unlike in Newtonian fluids^60^, the swimmer-enhanced effective diffusion and mixing will be highly anisotropic. Since in mucus the bacteria closely follow their own tracks, the diffusion perpendicular to the tracks will be mostly unaffected by bacterial swimming, while within the tracks it will be greatly enhanced.

## Methods

### Bacterial preparation

#### Bacillus subtilis

Strain 1085 (mutant strain that almost never tumbles) was grown in Terrific Broth (TB) at 30 °C in sealed vials under microaerobic conditions until an early stationary stage of concentration *c*_0_ ≈ 8 × 10^14^*m*^−3^ following^61^. Cells are then washed by centrifugation and suspended in TB. The cells swim in TB at a speed of (19 ± 5) *μm*/*s*, with the uncertainty given by the standard deviation of the distribution.

For flagella visualization, *Bacillus subtilis* bacteria, strain DS1919 (run-and-tumble), were grown overnight (≈16 *h*) in Luria-Bertani (LB) at 30° until late-logarithmic or early stationary phase. The flagella were stained by suspending a 1 *mL* drop of this broth with 2 *μL* of Alexa Fluor 488 C5-maleimide diluted in dimethyl sulfoxide at 5 *μg*/*mL*. Bacteria were incubated for 2 minutes at room temperature and washed three times in 1 *mL* of LB. This follows the guidelines of^62^. The average speed was (12 ± 2) *μm*/*s*, with the uncertainty being the standard deviation of the population.

#### *Escherichia coli*

The strains used were RP437 (run-and-tumble) and a smooth swimmer mutant strain CR20 (Δ*CheY*) expressing GFP (Green Fluorescent Protein) from a plasmid. The cells were grown overnight at 30 °C in Luria Broth (LB) plus the corresponding antibiotics until late-logarithmic or early stationary phase. Cells are then washed by centrifugation and suspended in LB.

### Visualization

The cells were visualized using an Olympus IX83 inverted microscope with a motorized stage, mounted on a Herzan TS-150 piezoelectric isolation platform. The bright field images were taken with a monochrome Prosilica GX 1660 camera (resolution of 1600 × 1200) at a frame rate of 30 and 50 frames per second (fps) using 20× and 60× magnification lens respectively. For the flagella visualization, we used a Hamamatsu ORCA-Flash4.0 V3 camera (resolution of 2048 × 2048) at 30 fps, with a 60× oil immersion objective.

### Cow cervical mucus

Mucus was collected from Holstein dairy heifers in estrus prior to artificial insemination. The reproductive tract (cervix and uterus) was gently palpated per rectum to aid in mucus flow from the cervix through the vaginal canal. Expressed mucus was collected into 50 *mL* conical tubes and immediately placed at 4 °C until used.

All procedures involving animals are reviewed and approved by the Pennsylvania State University Institutional Animal Care and Use Committee (protocol #200346584) and comply with the Guide for the Care and Use of Agricultural Animals in Agricultural Research and Teaching.

### Estimation of experimental shear rates

In our experiments, typical shear rates involved in the deposition of mucus are between 30 *s*^−1^ and 50 *s*^−1^. The rheological properties and also typical shear rates for mucus varies with shear stress and length scale. In addition, typical shear rates in cervical mucus also depend on the timing with respect to ovulation. Maximum physiological shear rates *in vivo* are estimated to be between 500 *s*^−1^ and 20000 *s*^−1^ for diverse sources of mucus (submaxillary, stomach, small intestine, colon, tracheobronchiolar, sputum, cervical and vaginal)^63^. These values range from one to three orders of magnitudes higher than the typical shear rates involved in the experiments here presented. Therefore, the shear-induced mucus anisotropy here found is relevant in the reproductive track. Moreover, the same experimental set up can be used to study other types of mucus.

We estimate the average shear rate of depositing mucus with a syringe and a needle as $\dot{\gamma }=4v/R \sim 33\,{s}^{-1}$, where *v* ~ 1 *mm*/*s* is the approximate velocity of extrusion and *R* = 0.12 *mm* is the radius of the needle.

In the parallel plates experiment, the maximal shear rate of the process will be $\dot{\gamma }=2v/H$, where $v \sim \frac{{R}_{2}-{R}_{1}}{t}$ is the approximate velocity of the liquid when moving from the configuration of the droplet of radius *R*_1_ ~ 150 *μm* and height *H*_1_ ~ 100 *μm* to a configuration of disk of height *H*_2_ = 10 *μm* confined between glass slides. If we model the volume of the droplet as the volume of a pyramid, then ${\rm{V}}{\rm{o}}{\rm{l}}{\rm{u}}{\rm{m}}{\rm{e}}=\pi {R}_{1}^{2}{H}_{1}\mathrm{/3}=\pi {R}_{2}^{2}{H}_{2}$ leads us to an expression for a typical value of *R*_2_. We estimate the squeezing time to be *t* ~ 0.5 *s*. Together, these magnitudes point to an approximate shear rate $\dot{\gamma } \sim 50\,{s}^{-1}$.

### SEM

A variable pressure field emission scanning electron microscope SIGMA VP-FESEM was used for acquisition of the cryo SEM micrographs. The samples were either a drop or a thread of mucus deposited on the metallic holder and immediately frozen in liquid Nitrogen. The mucus was stored at −80 °C and thawed 30 minutes prior to cryo SEM procedures. When stored at this temperature, the gel retained its original properties.

## Supplementary information

Supplementary Information

Supplementary video 1

Supplementary video 2

Supplementary video 3

Supplementary video 4
