# Supplementary material for: Mechanical shear controls bacterial penetration in mucus
Source: Sci Rep. 2019 Jul 4;9:9713. doi: 10.1038/s41598-019-46085-z (PMC6609767; doi:10.1038/s41598-019-46085-z)
Supplement: Supplementary file 1 — Supplementary Information [file 41598_2019_46085_MOESM1_ESM.pdf]

Supplementary Information: Mechanical shear controls bacterial penetration in mucus

Nuris Figueroa-Morales[1], Leonardo Dominguez-Rubio[1], Troy L. Ott[2], and Igor S. Aranson[1,*]

[1]Department of Biomedical Engineering, The Pennsylvania State University, University Park, PA 16802, USA
[2]Department of Animal Science and Center for Reproductive Biology and Health, The Pennsylvania State University, University Park, PA 16802, USA
[*]isa12@psu.edu

Flagella bending

Supplementary Figure S1 shows a sequence of snapshots revealing the bending of the flagella when the body is suddenly stopped in a shallow meniscus.

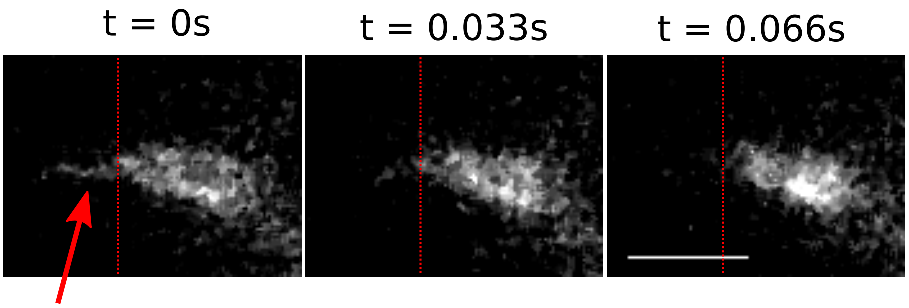

Figure S1. Flagella bending in a bacterium reversing direction. The flagella are initially at the left of the red line and as time goes the bundle bends out of plane, eventually crossing the red line, while the bacterium remains in place. The scale bar represents 5 μm.

Oscillations of Concentration

Supplementary Figure S2 shows the power spectrum of the average bacterial velocity along the main axis of mucus. It has a clear peak at $f = 0.3Hz$, evidencing a collaborative phenomenon of bacteria leaving the bouncing point together.

Rheology of mucus

The macrorheology of mucus was characterized by a shear sweep test using cone and plane geometry in a Discovery Hybrid Rheometer 3. The results are shown in Supplemmentary Figure S3. The liquid exhibits some shear thinning, as seen from the red curve for the complex viscosity. The storage modulus is higher than the loss modulus for the explored frequencies, reinforcing the evidence of predominantly elastic fluid. The quantitative results are in agreement with those of[1].

Suplementary Videos

Video1 Bacillus subtilis bacteria swimming in non-sheared mucus.

Video2 Bacillus subtilis bacteria invading mucus. A squeezed drop of mucus (left top corner) is invaded by Bacillus subtilis bacteria suspended in a squeezed drop of Terrific Broth (right bottom corner). The invasion takes place organized, with bacteria swimming radially.

Video3 180° reversal of the bacterial swimming direction in a mucus film. Bacillus subtilis bacteria swim towards the shallow part of the meniscus, invert their swimming direction and return with rare U-turns.

Video4 Bacillus subtilis bacterium reversing direction in a shallow meniscus. The flagella are stained and show their quick reorganization on the other side of the bacterium.

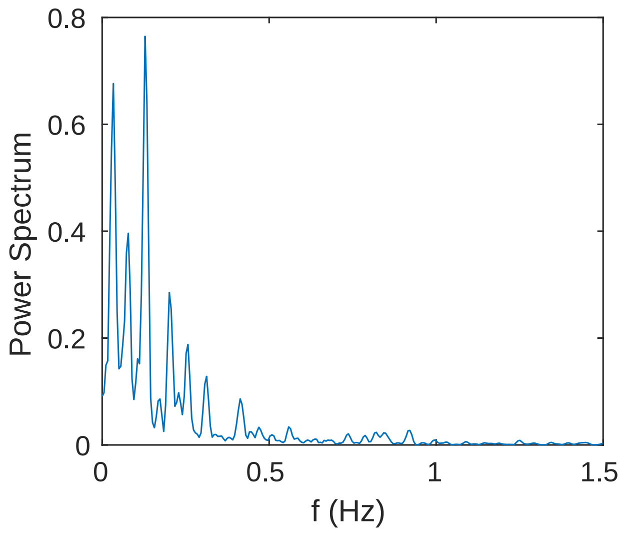

Figure S2. Power spectrum of average velocity of bacteria swimming in a meniscus of mucus. The main frequency is $f = 0.125Hz$.

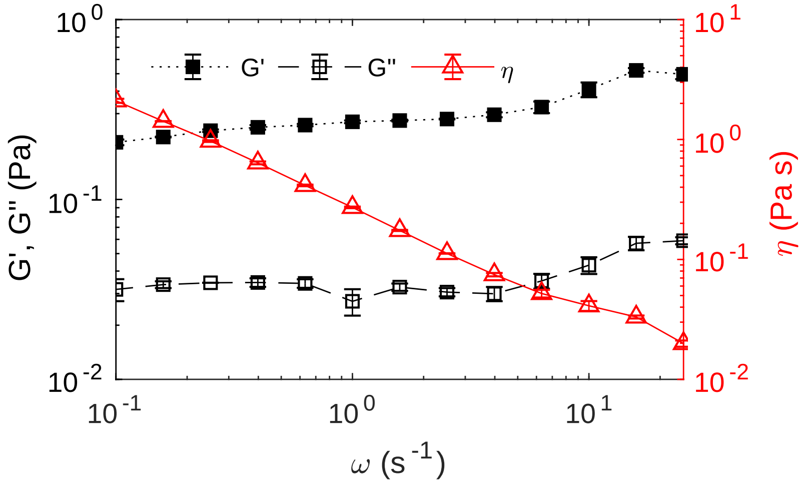

Figure S3. Mucus rheology. Results from a shear sweep test showing the complex viscosity (η) and the storage (G') and loss (G'') moduli.

References

1. Dawson, M., Wirtz, D. & Hanes, J. Enhanced viscoelasticity of human cystic fibrotic sputum correlates with increasing microheterogeneity in particle transport. *J. Biol. Chem.* **278**, 50393–50401 (2003).
